# *Journal of Patient-Reported Outcomes*: aims and scope

**DOI:** 10.1186/s41687-017-0009-2

**Published:** 2017-09-12

**Authors:** Dennis Revicki, David Feeny

**Affiliations:** 1 Outcomes Research, Evidera, Bethesda, MD USA; 2 Department of Economics, McMaster University, Hamilton, ON Canada

As founding Co-Editors-in-Chief of the *Journal of Patient-Reported Outcomes* (*JPRO*), we are excited to announce the launch of a new journal sponsored by the International Society for Quality of Life Research (ISOQOL). *JPRO* is intended to complement and extend ISOQOL’s existing journal, *Quality of Life Research*. Clearly, *Quality of Life Research* has a long and successful history of publishing research on methods and applications of health-related quality of life (HRQL) and patient-reported outcomes (PROs). Given increased HRQL and PRO research, and the wider application of these outcomes across a variety of health care and population health settings, there is a need for more publication outlets for scientifically sound PRO research.

The aims and scope of the *Journal of Patient-Reported Outcomes* focus on high quality research in five major areas: (1) PROs in clinical trials; (2) applications of PROs in clinical practice; (3) patient, family, community and public engagement in PRO research; (4) qualitative studies on the development and application of PROs; and (5) social and behavioral determinants of health and PRO measures. These focus areas are only some examples of the types of papers that are considered for publication in *JPRO*. *JPRO* is a peer-reviewed journal, with occasional special non-peer reviewed articles relevant to the journal’s aims and scope.

## Patient-reported outcomes in clinical trials

For many clinical trials, space limitations in clinical journals often preclude reporting on the HRQL and other PRO results in full. We encourage the publication of comprehensive analyses and reporting of evidence based on the use of PROs in clinical trials and other clinical studies. Authors of papers in *JPRO* are able to present and discuss new and more comprehensive evidence on treatment effects, on measurement properties, interpretation, and the performance of the PRO measures based on data from these clinical studies. The CONSORT extension on PROs will serve as a guide for reviewing the reporting of HRQL and PROs from clinical trials [1].

## Patient-reported outcomes in clinical practice

Increasingly, PROs are being implemented in clinical practice settings to inform patient management and for assessing the quality of health care. The application of PRO measures across health care systems and for use in real-world comparative effectiveness studies are of interest to *JPRO*. Methods for, and applications of, individual-level and group-level PRO applications from research studies are considered for publication. Applications such as quality improvement, benchmarking, patient experience with care, decision aids, communication, patient and provider satisfaction, adherence, patient management (including utilization of healthcare services and referral patterns), and other patient outcomes relying on PROs are considered for publication in *JPRO*.

## Patient, family, community, and public engagement

Patient, family, and community engagement in the planning and implementation of clinical studies and the evaluation of treatments and health care system interventions is growing rapidly [2]. The development and evaluation of methods for the meaningful engagement of patients and community participants in clinical and policy research relevant to PROs are considered for publication in *JPRO*. Methods and applications to engage patients, family members, and members of the general public in the process of PRO-related research, and the evaluation of the effects of such engagement, are of interest. Similarly research and evaluations of engaging patients and the general public related to PRO policy formulation and clinical guidelines are welcome, as is participatory action research that use PROs.

## Qualitative and mixed methods studies on the development and application of PROs

Qualitative methods are frequently used in the development and evaluation of new and existing PRO measures, and increasingly used in conjunction with clinical trials. These studies increase understanding of the effects and burden of illness, and provide more complete understanding of the effects of treatment from the patient perspective. Papers reporting on these activities, the assessment of the usefulness of various qualitative methods for these purposes, and improvements in qualitative methods are considered for publication by *JPRO*. Studies that give patients a voice and report patient stories using PROs are also welcome.

## Social and behavioral determinants of health using PROs

Studies reporting on cross-sectional and longitudinal observational studies examining the social and behavioral determinants of health with PRO measures are considered for publication, including studies of both general and chronic disease populations. Such studies might examine the antecedents of health-related outcomes such as social support, health-related behaviors, and patient adherence. *JPRO* welcomes submissions that report on studies that include HRQL outcomes and other PROs.

In addition, *JPRO* considers review articles, brief communications, commentaries, editorials, and reviews of recent books and software advances relevant to the stated aims and scope of the journal, although in general, *JPRO* does not publish case reports, papers reporting study designs, pilot studies, and feasibility studies.

As the inaugural Co-Editors-In-Chief, we are looking forward to the successful development of *JPRO* and to encouraging sound research and science related to HRQL and PRO applications in clinical trials and clinical practice. Over the past 25 years, we have seen continued and exciting developments in HRQL and PRO research and methods, and we hope to encourage further the reporting of these methods and results in *JPRO*. To paraphrase a historic moment in cinema, “this is the beginning of a beautiful journal”. We look forward to your support and participation in developing and advancing the new journal.
